# Correction: Real-Time Predictions of Reservoir Size and Rebound Time during Antiretroviral Therapy Interruption Trials for HIV

**DOI:** 10.1371/journal.ppat.1005778

**Published:** 2016-07-13

**Authors:** The PLOS Pathogens Staff

## Notice of Republication

This article was republished on June 29, 2016, to correct errors in Table 1 that were introduced during the typesetting process. The publisher apologizes for the errors. Please download this article again to view the correct version. The originally published, uncorrected article and the republished, corrected articles are provided here for reference.

## Supporting Information

S1 File Originally published, uncorrected article. (PDF)

S2 File Republished, corrected article. (PDF)
