# Correction: Real-Time Predictions of Reservoir Size and Rebound Time during Antiretroviral Therapy Interruption Trials for HIV

**DOI:** 10.1371/journal.ppat.1006488

**Published:** 2017-07-06

**Authors:** Alison L. Hill, Daniel I. S. Rosenbloom, Edward Goldstein, Emily Hanhauser, Daniel R. Kuritzkes, Robert F. Siliciano, Timothy J. Henrich

S3 Text is missing the reference list. The missing references are as follows:

[1]Eriksson S, Graf EH, Dahl V, Strain MC, Yukl SA, Lysenko ES, et al. Comparative Analysis of Measures of Viral Reservoirs in HIV-1 Eradication Studies. PLoS Pathog. 2013 Feb;9(2):e1003174. Available from: http://dx.doi.org/10.1371/journal.ppat.1003174.

[2]Crooks AM, Bateson R, Cope AB, Dahl NP, Griggs MK, Kuruc JD, et al. Precise Quantitation of the Latent HIV-1 Reservoir: Implications for Eradication Strategies. Journal of Infectious Diseases. 2015 Apr;p. jiv218. Available from: http://jid.oxfordjournals.org/content/early/2015/04/15/infdis.jiv218.

[3]Siliciano JD, Kajdas J, Finzi D, Quinn TC, Chadwick K, Margolick JB, et al. Long-term follow-up studies con_rm the stability of the latent reservoir for HIV-1 in resting CD4+ T cells. Nat Med. 2003 Jun;9(6):727{728. Available from: http://dx.doi.org/10.1038/nm880.

[4]Hill AL, Rosenbloom DIS, Fu F, Nowak MA, Siliciano RF. Predicting the outcomes of treatment to eradicate the latent reservoir for HIV-1. Proc Natl Acad Sci USA. 2014 Sep;111(37):13475{13480. Available from: http://www.pnas.org/content/111/37/13475.

[5]Ho YC, Shan L, Hosmane NN, Wang J, Laskey SB, Rosenbloom DIS, et al. ReplicationCompetent Noninduced Proviruses in the Latent Reservoir Increase Barrier to HIV-1 Cure. Cell. 2013 Oct;155(3):540{551. PMID: 24243014.

[6]Archin NM, Vaidya NK, Kuruc JD, Liberty AL, Wiegand A, Kearney MF, et al. Immediate antiviral therapy appears to restrict resting CD4+ cell HIV-1 infection without accelerating the decay of latent infection. Proc Natl Acad Sci USA. 2012 Jun;109(24):9523{9528.

[7]Shen L, Peterson S, Sedaghat AR, McMahon MA, Callender M, Zhang H, et al. Dose-response curve slope sets class-speci_c limits on inhibitory potential of anti-HIV drugs. Nature medicine. 2008;14(7):762766.

[8]Jilek BL, Zarr M, Sampah ME, Rabi SA, Bullen CK, Lai J, et al. A quantitative basis for antiretroviral therapy for HIV-1 infection. Nature Medicine. 2012 Feb;18(3):446{451. Available from: http://www.nature.com/nm/journal/v18/n3/full/nm.2649.html.

[9]Henrich TJ, Hu Z, Li JZ, Sciaranghella G, Busch MP, Keating SM, et al. Long-Term Reduction in Peripheral Blood HIV-1 Reservoirs Following Reduced-Intensity Conditioning Allogeneic Stem Cell Transplantation. Journal of Infectious Diseases. 2013 Jun;207(11):1694{1702. Available from: http://jid.oxfordjournals.org/content/early/2013/02/28/infdis.jit086.

[10]Sedaghat AR, Siliciano JD, Brennan TP, Wilke CO, Siliciano RF. Limits on Replenishment of the Resting CD4+ T Cell Reservoir for HIV in Patients on HAART. PLoS Pathog. 2007;3(8):e122. Available from: http://dx.plos.org/10.1371/journal.ppat.0030122.

[11]Nettles R, Kie_er TL, Kwon P, Monie D, Han Y, Parsons T, et al. Intermittent HIV-1 viremia (blips) and drug resistance in patients receiving HAART. JAMA. 2005 Feb;293(7):817{829. Available from: http://dx.doi.org/10.1001/jama.293.7.817.

[12]Dinoso JB, Kim SY, Wiegand AM, Palmer SE, Gange SJ, Cranmer L, et al. Treatment intensi_cation does not reduce residual HIV-1 viremia in patients on highly active antiretroviral therapy. Proc Natl Acad Sci USA. 2009;106(23):9403.

The corrected S3 Text is provided here.

## Supporting information

S3 Text Estimating the size and source of the reservoir post-transplant. (PDF)
