# Supplementary material for: Real-Time Predictions of Reservoir Size and Rebound Time during Antiretroviral Therapy Interruption Trials for HIV
Source: PLoS Pathog. 2016 Apr 27;12(4):e1005535. doi: 10.1371/journal.ppat.1005535 (PMC4847932; doi:10.1371/journal.ppat.1005535)
Supplement: S1 Text — (PDF) [file ppat.1005535.s001.pdf]

Estimating reservoir size with uncertainty in viral dynamics parameters

Supplementary Information for

Real-time predictions of reservoir size and rebound time during antiretroviral therapy interruption trials for HIV

Alison L. Hill[1,*], Daniel I. S. Rosenbloom[2], Edward Goldstein[3], Emily Hanhauser[4], Daniel R. Kuritzkes[4], Robert F. Siliciano[5,†], and Timothy J. Henrich[6,†]

[1]Program for Evolutionary Dynamics, Department of Mathematics, Department of Organismic and Evolutionary Biology, Harvard University, Cambridge, MA, USA
[2]Department of Biomedical Informatics, Columbia University Medical Center, New York, NY, USA
[3]Center for Communicable Disease Dynamics, Department of Epidemiology, Harvard T.H. Chan School of Public Health, Boston, MA, USA
[4]Division of Infectious Diseases, Brigham and Women's Hospital, Harvard Medical School, Boston, MA, USA
[5]Department of Medicine, Johns Hopkins University School of Medicine and Howard Hughes Medical Institute, Baltimore, MD, USA
[6]Division of Experimental Medicine, Department of Medicine, University of California, San Francisco, CA, USA
[†]These senior authors contributed equally to the work
[*]To whom correspondence should be addressed: alhill@fas.harvard.edu

Numerical Methods

In the Methods (Eq. 1), we showed that if rebound has still not occurred by time τ after treatment interruption, then the posterior probability distribution for the reduction efficacy q is given by

$$P_{post}(q \mid t_r > \tau) = \frac{S(\tau \mid q)P(q)}{\int_q S(\tau \mid q)P(q)dq}. \quad (1)$$

Here $S(t \mid q)$ is the model prediction for the fraction of patients who would not have rebounded by a time t for a given q. $P(q)$ is the prior probability distribution for our existing knowledge (after reservoir reduction but before treatment interruption) about the likelihood of a given q value being the true value; and would generally be based on information from experimental assays.

Although not explicitly written here, the function $S(t \mid q)$ depends on a set of input parameters, which we call Θ, used in the stochastic viral dynamics model to predict rebound times, and is therefore more correctly written as $S(t \mid q, \Theta)$. For most of the results presented, we have assumed that these parameter values are known, and have used the best estimated values reported in [1].

However, in reality there is some uncertainty about the exact value of each parameter. There are a range of parameter values that are consistent with the data sources from which they were estimated, though some values may be more probable than others. Therefore instead of using a point estimate for each parameter value, we can construct a probability distribution for the values. Like $P(q)$ describes our existing knowledge about the reservoir size before treatment interruption, $P(\Theta)$ describes this knowledge for the viral dynamic parameters.

Uncertainty about parameter values alters our interpretation of rebound times. The time of rebound now not only helps inform our estimate of the reservoir size, but it also informs our estimate of the viral dynamics parameters. A long rebound time could be consistent with a small reservoir size, or with favorable parameters, such as a low activation rate of latently infected cells or a long latent cell lifespan. The posterior probability distribution now describes both q and Θ

$$P_{post}(q, \Theta \mid t_r > \tau) = \frac{S(\tau \mid q, \Theta)P(q)P(\Theta)}{\int_q S(\tau \mid q, \Theta)P(q, \Theta)dq}. \tag{2}$$

It is no longer practical to evaluate Eq. 2 by simulating the model of rebound dynamics for many patients at many q and Θ values and then numerically integrating over the results. The simulation is computationally intensive even for a single set of parameters and the resulting five-dimensional parameter space is intractable. Instead, we employed a Markov Chain Monte Carlo method to draw samples from $S(\tau \mid q, \Theta)P(q)P(\Theta)$. We calculate $S(t \mid q, \Theta)$ using a highly-accurate numerical approximation developed and tested in our previous paper [1]. Samples were generated using the Metropolis-Hastings methods with a symmetric (log) Gaussian proposal distribution for each parameter, with a standard deviation of 0.5 for q and half the standard deviation of the prior for the other parameters. 10,000 samples were taken and probability densities were constructed using all samples after a 1000-step burn-in.

Parameter Distributions

In our previous work, we concluded that model outcomes depend on a small number of parameters, which could be estimated from existing clinical data [1]. Our numerical method to calculate $S(\tau \mid q, \Theta)$ requires only these parameters for input. Specifically, the half-life of the pool of latently infected cells ($t_{\frac{1}{2}}$)is estimated from studies of LR decay [2, 3], and the activation rate of latent cells (A) and the viral growth rate (r) can both be estimated from studies of supervised cART interruption [4, 5]. A fourth parameter, the probability that a single activated cell manages to establish a growing infection, is less certain and is the subject of both *in vitro* [6] and population-genetic research [7, 8, 9]. It is determined both by the viral fitness and by the distribution of the number of new infections caused by a single infected cell [10]. A higher variance-to-mean ratio (ρ) for this distribution means there is more noise in the viral dynamics and a lower establishment probability.

We use prior distributions for these parameters that were constructed from the cited data sources (Table S1.1). All details of the parameter estimation are given in the previous manuscript [1]. The one change we made from previous work is for distribrution for the the activation rate of latent cells, A. This parameter is the total rate at which cells exit the reservoir before reservoir-reducing therapy, and so is the product of the number of cells present, and the per-cell activation rate. Variation in this parameter therefore includes both variation in the pre-latency-reversing-therapy reservoir size and in the per-cell activation rate. Since this reservoir size is measurable

in an individual patient, it is not unknown variation, and does not need to be incorporated into this distribution. Because reservoir sizes have about a 0.5-log standard deviation between infected individuals (200-fold variation between central 95% of individuals [11]), we subtract this variation and reduce the standard deviation of A from 1 (as per [1]) to 0.5. We assumed that each parameter varies independently of the others, so that the joint prior distribution is the product of the individual distributions: $P(\Theta) = P(\delta)P(A)P(r)P(\rho)$. Note that these distributions are likely overestimates for the true unmeasurable variation between individual patients in viral dynamics. They more likely represent limitations of current experimental techniques and statistical inference methods to determine these parameters, and will likely become more narrow as better estimates are produced.

Parameter	Symbol	Estimation Method	Source	Best Estimate	Distribution*
LR decay rate	δ	Long-term ART ($\delta = \ln(2)/\tau_{1/2}$)	[2, 3]	5.2×10^{-4} d^{-1}	$\delta \sim \mathcal{N}(5.2, 1.6) \times 10^{-4}$ d^{-1}
LR exit rate	A	Viral rebound after ART interruption	[4, 5]	57 cells d^{-1}	$\log_{10}(A) \sim \mathcal{N}(1.76, 0.5)$
Growth rate	r			0.4 d^{-1}	$\log_{10}(r) \sim \mathcal{N}(-0.40, 0.19)$
Variance-to-mean ratio of viral offspring distribution	ρ	Population genetic modeling; *in vitro* virology	[6-10]	0.069	$\log_{10}(\rho) \sim \mathcal{N}(1, 1.175)$

Table S1.1. Estimated values for the key parameters of the stochastic viral dynamics model
*Notation $X \sim \mathcal{N}(\mu, \sigma)$ means that X is a random variable drawn from a normal distribution with mean μ and standard deviation σ.

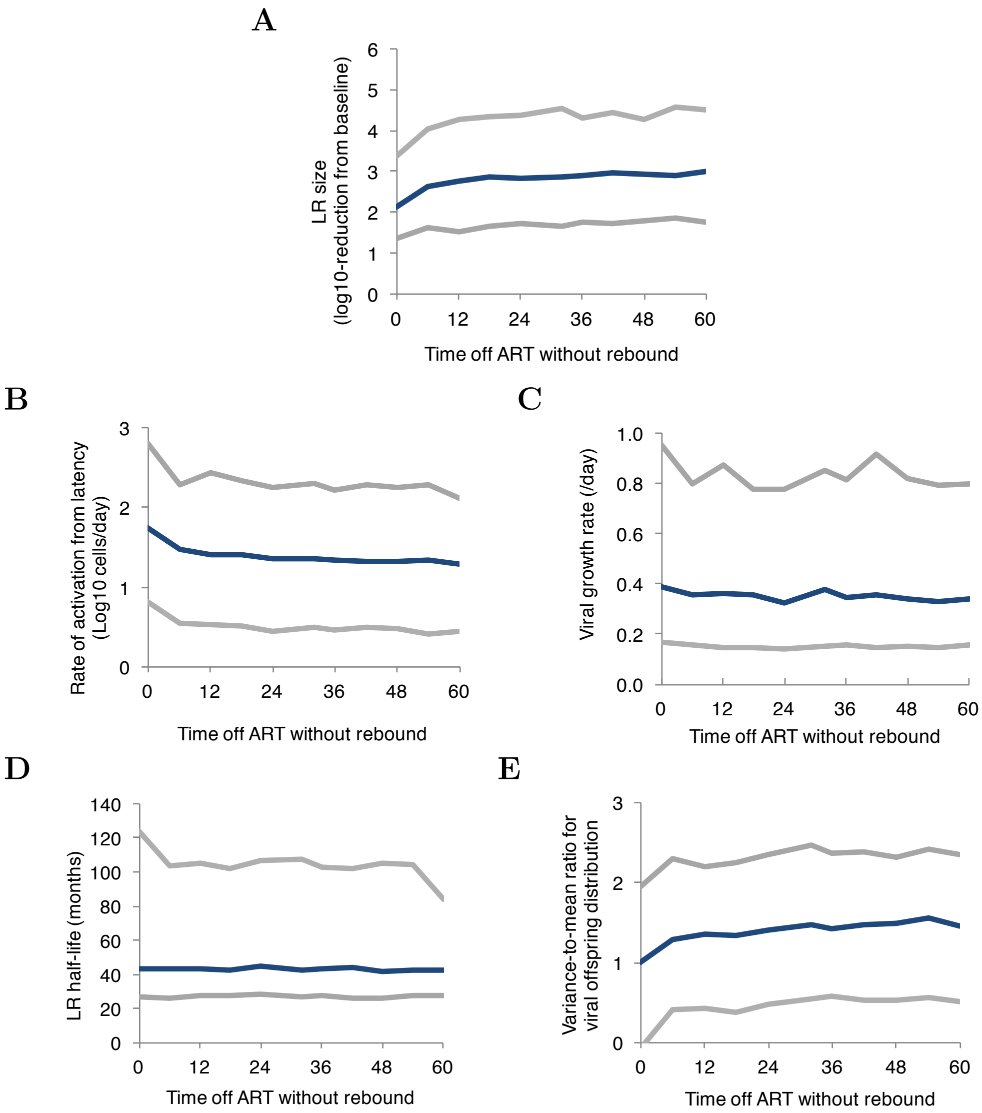

Figure S1.1. Interpreting the outcomes of treatment interruption when the reservoir reduction is unknown and there is uncertainty in viral dynamics parameters. The Bayesian approach described above was used to update the estimates for the reservoir size and the viral dynamics parameters based on the current time off treatment *without* rebound. The posterior median (dark blue line) and 95% credible intervals (light grey lines) are shown for A) The reservoir size, B) The rate at which latent cells become activated, A, C) The viral growth rate, r, D) The half-life of the pool of latently infected cells ($t_{\frac{1}{2}}$, or $\ln(2)/\delta$), and E) The variance-to-mean ratio of the viral offspring distribution, which determines the single activated cell manages to establish a growing infection. The prior on the reservoir size is the same as in Figure 2a. The priors on the viral dynamics parameters are the distributions listed in Table S1.1. Compared to Figure 2c, including uncertainty in viral dynamics parameters reduces the information gained about reservoir size as the time off treatment without rebound increases, and leads to decreases in estimates for the parameters that promote viral rebound (A, r, $t_{\frac{1}{2}}$), and decreases in those that hinder viral rebound (ρ). Because A and ρ have much greater effects on rebound times than r and $t_{\frac{1}{2}}$, estimates for these parameters change more over time.

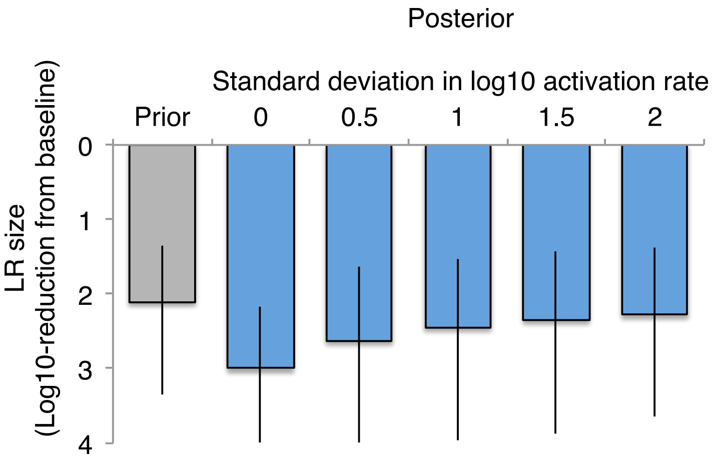

Figure S1.2. Posterior estimates for reservoir size in the presence of increasing uncertainty in the latent cell activation rate The Bayesian approach described above was used to update the estimates for the reservoir size based on the current time off treatment *without* rebound. In each case, the prior probability distribution for the latent cell activation rate A was assumed to be of the form $\log_{10}(A) \sim \mathcal{N}(1.76, \sigma)$, where the log10-standard deviation σ varied from a low of 0.5 (results in Figure S1.1) to a high of 2. We also include the case where all parameters are known exactly ($\sigma = 0$, compare to Figure 2c). For each case, the posterior median reservoir reduction (height of blue bars) and 95% credible intervals (error bars) were calculated assuming 6 months had passed without viral rebound. The prior on the reservoir size is the same as in Figure 2a (also shown with grey bar). The priors on the other viral dynamics parameters are the distributions listed in Table S1.1, except for the case with $\sigma = 0$, in which all parameters were fixed at the "Best Estimate" values. Larger uncertainty in the value of A leads to less information about the reservoir size gained from observations during treatment interruption trials. In other words, the posterior distribution for q looks more similar to the prior distribution For σ values of 0.5, 1, 1.5 and 2, the fold-difference in A between the central 95% of values is 90, 8000, 8×10^5, and 7×10^7, respectively, demonstrating that the approach only becomes uninformative for extremely large uncertainty in parameter values.

References

[1] Hill AL, Rosenbloom DIS, Fu F, Nowak MA, Siliciano RF. Predicting the outcomes of treatment to eradicate the latent reservoir for HIV-1. Proc Natl Acad Sci USA. 2014 Sep;111(37):13475–13480. Available from: http://www.pnas.org/content/111/37/13475.

[2] Siliciano JD, Kajdas J, Finzi D, Quinn TC, Chadwick K, Margolick JB, et al. Long-term follow-up studies confirm the stability of the latent reservoir for HIV-1 in resting CD4+ T cells. Nat Med. 2003 Jun;9(6):727–728. Available from: http://dx.doi.org/10.1038/nm880.

[3] Crooks AM, Bateson R, Cope AB, Dahl NP, Griggs MK, Kuruc JD, et al. Precise Quantitation of the Latent HIV-1 Reservoir: Implications for Eradication Strategies. Journal of Infectious Diseases. 2015 Apr;p. jiv218. Available from: http://jid.oxfordjournals.org/content/early/2015/04/15/infdis.jiv218.

[4] Luo R, Piovoso MJ, Martinez-Picado J, Zurakowski R. HIV Model Parameter Estimates from Interruption Trial Data including Drug Efficacy and Reservoir Dynamics. PLoS ONE. 2012 Jul;7(7):e40198. Available from: http://dx.doi.org/10.1371/journal.pone.0040198.

[5] Ruiz L, Martinez-Picado J, Romeu J, Paredes R, Zayat MK, Marfil S, et al. Structured treatment interruption in chronically HIV-1 infected patients after long-term viral suppression. AIDS. 2000;14(4):397.

[6] Singh A, Razooky B, Cox CD, Simpson ML, Weinberger LS. Transcriptional bursting from the HIV-1 promoter is a significant source of stochastic noise in HIV-1 gene expression. Biophysical journal. 2010;98(8):L32–4.

[7] Pennings PS. Standing Genetic Variation and the Evolution of Drug Resistance in HIV. PLoS Comput Biol. 2012 Jun;8(6):e1002527. Available from: http://dx.doi.org/10.1371/journal.pcbi.1002527.

[8] Pennings PS, Kryazhimskiy S, Wakeley J. Loss and recovery of genetic diversity in adapting populations of HIV. PLoS Genetics. 2014;10(1):e1004000.

[9] Rouzine IM, Coffin JM. Linkage disequilibrium test implies a large effective population number for HIV in vivo. Proc Natl Acad Sci USA. 1999;96(19):10758–10763.

[10] Rouzine IM, Razooky BS, Weinberger LS. Stochastic variability in HIV affects viral eradication. Proceedings of the National Academy of Sciences. 2014 Sep;111(37):13251–13252. Available from: http://www.pnas.org/content/111/37/13251.

[11] Eriksson S, Graf EH, Dahl V, Strain MC, Yukl SA, Lysenko ES, et al. Comparative Analysis of Measures of Viral Reservoirs in HIV-1 Eradication Studies. PLoS Pathog. 2013 Feb;9(2):e1003174. Available from: http://dx.doi.org/10.1371/journal.ppat.1003174.
