# Supplementary material for: Real-Time Predictions of Reservoir Size and Rebound Time during Antiretroviral Therapy Interruption Trials for HIV
Source: PLoS Pathog. 2016 Apr 27;12(4):e1005535. doi: 10.1371/journal.ppat.1005535 (PMC4847932; doi:10.1371/journal.ppat.1005535)
Supplement: S2 Text — (PDF) [file ppat.1005535.s002.pdf]

Estimating reservoir re-seeding during viral rebound

Supplementary Information for

Real-time predictions of reservoir size and rebound time during antiretroviral therapy interruption trials for HIV

Alison L. Hill[1,*], Daniel I. S. Rosenbloom[2], Edward Goldstein[3], Emily Hanhauser[4], Daniel R. Kuritzkes[4], Robert F. Siliciano[5,†], and Timothy J. Henrich[6,†]

[1]Program for Evolutionary Dynamics, Department of Mathematics, Department of Organismic and Evolutionary Biology, Harvard University, Cambridge, MA, USA
[2]Department of Biomedical Informatics, Columbia University Medical Center, New York, NY, USA
[3]Center for Communicable Disease Dynamics, Department of Epidemiology, Harvard T.H. Chan School of Public Health, Boston, MA, USA
[4]Division of Infectious Diseases, Brigham and Women's Hospital, Harvard Medical School, Boston, MA, USA
[5]Department of Medicine, Johns Hopkins University School of Medicine and Howard Hughes Medical Institute, Baltimore, MD, USA
[6]Division of Experimental Medicine, Department of Medicine, University of California, San Francisco, CA, USA
[†]These senior authors contributed equally to the work
[*]To whom correspondence should be addressed: alhill@fas.harvard.edu

To estimate the number of latently infected cells that could be generated if viral rebound goes undetected for a few days, we first simulated realistic viral dynamics, then used an established empirical relationship to relate this to reservoir size. To simulate viral loads and CD4 counts, we used an extension of the basic viral dynamics model [1] that includes a density-dependent death rate of infected cells, representing CTL killing that is stimulated by the infection level. We numerically integrated the following system of differential equations:

$$
\begin{aligned}
\dot{C} &= \lambda - \beta C V - d_C C \\
\dot{I} &= \beta(1-f) C V - \left(d_I + q \frac{I}{s+I} \right) I \\
\dot{V} &= kI - d_V V \\
\dot{L} &= \beta f C V - d_L L.
\end{aligned}
\tag{1}
$$

Here the variable C is the concentration of uninfected CDT cells, I the actively infected cells, and L latently infected cells. V is the viral load. Uninfected cells are produced at rate λ, die at rate d_C, and are infected by virus at rate β. Infected cells become active with probability f and

start producing virus at rate k, or, become latent. Infected cells die at a baseline rate d_I and an additional density-dependent rate, which has a half-max when $I = s$ and a maximum rate of q. Latent cells die at rate d_L. We used parameters $\lambda = 10$, $\beta = 1.8 \times 10^{-7}$, $k = 10^5$, $f = 10^{-4}$, $d_C = 0.01$, $d_I = 0.5$, $q = 1$, $s = 10^6$, $d_L = 0$, $d_V = 20$, and initial conditions $C(0) = \lambda/d_C$, $V(0) = 0.1$, $I(0) = (d_V/k)V(0)$, $L(0) = 0$. The value of $V(0)$ has a negligible influence on the results and the value of $L(0)$ does not matter if we interpret the estimates of reservoir size after re-seeding to be the increase from the value before ART-interruption. Reservoir decay is very slow and not relevant on the timescale of rapid rebound. With these parameters the exponential growth rate is 0.4/day, the set-point is $\approx 4 \times 10^4$ c/ml, and it takes 19 days for viral load to reach 200 c/ml.

Using observed values for viral load ($V(t)$) and CD4 counts ($C(t)$) during acute infection, Archin et al [2] showed that the predicted number of latently infected cells that this model, which would be

$$L_{pred}(t) = L(0)e^{-d_L t} + e^{-d_L t} f\beta \int_0^t e^{d_L \tau} V(\tau)C(\tau)d\tau, \tag{2}$$

was correlated strongly ($r = 0.65$) with the observed latent cells, measured by a viral co-culture assay ($L_{obs}(t)$). The observed log-scale regression was

$$\log_{10}(L_{obs}(t)) = 0.35 \log_{10}\left(\frac{L_{pred}(t)}{C(t)} 10^6 \frac{1}{10^{14} f\beta}\right) + 0.35. \tag{3}$$

We first used the model in Eq. (1) to generate $L_{pred}(t)$, then used Eq. (3) to generate $L_{obs}(t)$. The first scaling of L_{pred} is to convert from a concentration to a frequency of infectious units per million (IUPM), and the second was used to divide out unknown parameters when empirical viral load and CD4 values were used to estimate L_{pred} in Ref. [2]. To examine a worst-case scenario for re-seeding, we assume that viral load is just below the detection limit at one sampling time-point and isn't detected until the next time-point. Therefore we set t equal to the time until viral load is detectable (which depends on the assay detection limit) plus the time between sampling points. Both the assay detection limit and the sampling frequency are varied for the presented results.

References

[1] Burg D, Rong L, Neumann AU, Dahari H. Mathematical modeling of viral kinetics under immune control during primary HIV-1 infection. Journal of Theoretical Biology. 2009 Aug;259(4):751–759. Available from: http://www.sciencedirect.com/science/article/pii/S0022519309001702.

[2] Archin NM, Vaidya NK, Kuruc JD, Liberty AL, Wiegand A, Kearney MF, et al. Immediate antiviral therapy appears to restrict resting CD4+ cell HIV-1 infection without accelerating the decay of latent infection. Proc Natl Acad Sci USA. 2012 Jun;109(24):9523–9528.
