# Supplementary material for: Real-Time Predictions of Reservoir Size and Rebound Time during Antiretroviral Therapy Interruption Trials for HIV
Source: PLoS Pathog. 2016 Apr 27;12(4):e1005535. doi: 10.1371/journal.ppat.1005535 (PMC4847932; doi:10.1371/journal.ppat.1005535)
Supplement: S3 Text — (PDF) [file ppat.1005535.s003.pdf]

Estimating the size and source of the reservoir post-transplant

Supplementary Information for

Real-time predictions of reservoir size and rebound time during antiretroviral therapy interruption trials for HIV

Alison L. Hill[1,*], Daniel I. S. Rosenbloom[2], Edward Goldstein[3], Emily Hanhauser[4], Daniel R. Kuritzkes[4], Robert F. Siliciano[5,†], and Timothy J. Henrich[6,†]

[1]Program for Evolutionary Dynamics, Department of Mathematics, Department of Organismic and Evolutionary Biology, Harvard University, Cambridge, MA, USA
[2]Department of Biomedical Informatics, Columbia University Medical Center, New York, NY, USA
[3]Center for Communicable Disease Dynamics, Department of Epidemiology, Harvard T.H. Chan School of Public Health, Boston, MA, USA
[4]Division of Infectious Diseases, Brigham and Women's Hospital, Harvard Medical School, Boston, MA, USA
[5]Department of Medicine, Johns Hopkins University School of Medicine and Howard Hughes Medical Institute, Baltimore, MD, USA
[6]Division of Experimental Medicine, Department of Medicine, University of California, San Francisco, CA, USA
[†]These senior authors contributed equally to the work
[*]To whom correspondence should be addressed: alhill@fas.harvard.edu

Summary

The observed viral rebound after months of antiretroviral-free remission in both Boston patients suggests that latently infected cells were present. Two sources are possible for these cells: host cells that survived chemotherapy and graft-vs-host disease, and donor cells that later became infected and entered latency. Here we detail the methods used to estimate the range of latently infected cells that could plausibly come from each source.

Remaining reservoir in host cells

The frequencies of HIV DNA positive cells pre-transplant were 144 and 96 per 10^6 PBMCs for Patients A and B, respectively. The times between when these reservoir measurement was made and when cART was interrupted were 1266 and 625 days. Residual host cells were detected at a frequency of less than 10^{-5} among all PBMC in both patients. Eriksson et al [?] showed that the ratio of PBMCs containing HIV DNA to the number of resting CD4$^+$ T cells that produced infectious HIV in a co-culture system was near 100:1 for the vast majority of patients (see their

Fig 2A), with around 95% of patients falling between 10:1 and 1000:1. The latent reservoir decays with a half-life that has been estimated at 44 months [?, ?, ?], which corresponds to an exponential decay rate, which we call d_L, of 5.2×10^{-4} per day. This can be used to factor in the reservoir reduction that would be expected between transplant and treatment interruption just due to the natural finite lifespan of these cells. Combining all this information, we can estimate the IUPM (HIV^+ cells per 10^6 $CD4^+$ T cells) for Patient A:

$$\begin{aligned} IUPM^A_{host} &\approx (\text{Initial frequency HIV DNA}^+ \text{ cells}) \times (\text{re-activateable cells per HIV DNA}^+ \text{ cell}) \\ &\quad \times (\text{Final \% chimerism }) \times (\text{Fraction surviving decay}) \\ &\approx \left(\frac{144 \text{ HIV DNA}^+ \text{ PBMC}}{10^6 \text{ host PBMC}}\right) \times \left(\frac{1\text{IUPM}}{\left(\frac{100 \text{ HIV DNA}^+ \text{ PBMC}}{10^6 \text{ PBMC}}\right)}\right) \times \left(\frac{1 \text{ host PBMC}}{10^5 \text{ PBMC}}\right) \\ &\quad \times e^{-5.2 \times 10^{-4} \text{days}^{-1} \times \text{ 1266 days}} \\ &\approx 7.5(0.75, 75) \times 10^{-6} \end{aligned} \tag{1}$$

where the range includes variation in the observed ratio of HIV DNA to productive infection. With the same method, we get $IUPM^B_{host} \approx 6.9$ (0.69, 69) $\times 10^{-6}$.

More recent work has shown that co-culture may miss some resting $CD4^+$ T cells containing integrated, replication-competent HIV. These cells, while they fail to lead to viral outgrowth in co-culture, are in principle capable of initiating viral replication under physiologic conditions providing repeated antigenic stimulation. Ho et al [?] (their Table S3) found that the frequency of predicted intact proviruses among PBMCs containing HIV DNA was 3.7% (95% confidence interval 0.5%-14.2%). To use these results we also have to take into account that Ho et al measured frequencies of HIV DNA in resting $CD4^+$ cells, while Henrich et al measured frequencies in all PBMC; which can be done using the fact that there are on average 4-fold more HIV DNA^+ cells in purified resting $CD4^+$ cells than in PBMC [?]. Using this range we get a modified estimate of

$$\begin{aligned} IUPM'^A_{host} &\approx \left(\frac{144 \text{ HIV DNA}^+ \text{ PBMC}}{10^6 \text{ host PBMC}}\right) \times \left(\frac{\left(\frac{4 \text{ HIV}^+ \text{ DNA}^+ \text{ CD4}^+ \text{ TC}}{10^6 \text{ CD4}^+ \text{ TC}}\right)}{\left(\frac{1 \text{ HIV DNA}^+ \text{ PBMC}}{10^6 \text{ PBMC}}\right)}\right) \\ &\quad \times \left(\frac{0.037 \text{ HIV}^{intact} \text{ CD4}^+ \text{ TC}}{1 \text{ HIV}^+ \text{ DNA}^+ \text{ CD4}^+ \text{ TC}}\right) \times \left(\frac{1 \text{ host PBMC}}{10^5 \text{ PBMC}}\right) \times e^{-5.25 \times 10^{-4} \text{days}^{-1} \text{ 1266 days}} \\ &\approx 110(15, 420) \times 10^{-6} \end{aligned} \tag{2}$$

where the range includes variation in the observed ratio of HIV DNA to intact provirus. With the same method, we get $IUPM'^B_{host} \approx 103$ (14, 390) $\times 10^{-6}$.

We can combine the estimates using frequencies from co-culture assays and from intact provirus to get an overall range. For our central estimate, we take the best-estimated value from co-culture assays, and for the maximum and minimum we take the more extreme value from each range. We therefore get that $IUPM^A_{host} \approx 7.5$ (0.75, 420) $\times 10^{-6}$ and $IUPM^B_{host} \approx 6.9$ (0.69, 390) $\times 10^{-6}$.

New infection of donor cells

We employ two different methods to estimate the number of cells that could have been newly infected and entered into latency in each patient over the course of transplant. We use what we believe to be the more reliable estimate from Method 1 as a central value, and then include the range from Method 2.

Method 1:

The rate of entry of cells into latency during cART can be estimated using the same approach as for acute infection (S2 Text Equations 2 and 3), which takes empirically observed viral load values over time and returns a predicted reservoir size in units of IUPM [**?**]. This method side-steps the need to directly know the infection rate and the fraction of newly infected cells that revert to latency, and instead estimated these parameters to within a proportionality by empirically observing reservoir sizes along with viral loads. To use this method, we must adjust for differences in parameters values between stages of infection (acute untreated vs chronic treated). The most important difference is the dramatically reduced infection rate (β) during cART. The factor by which single and combination antiretroviral drugs reduce viral infectivity has previously been reported for a large panel of drugs in [**?**, **?**], and the smallest reduction reported for a three drug combination was around 100-fold. To consider a worst-case scenario for new infection, we therefore take $\beta \to 10^{-2}\beta$ when calculating L_{pred} (S2 Text Eq. 2). Although no errors are given on the estimates for reservoir size reported by this method [**?**], visual inspection suggests that variation from this relationship by up to 1 log in either direction occurs in the patient population.

We describe $V(t)$ and $C(t)$ in S2 Text Eq.2 by a set of discrete values derived from viral load and CD4 cell count measurements at fixed time points. For simplicity, we assume that between observations, the remains at the value of the most recent test. Therefore it takes on the value V_i or C_i for a time period from t_i to t_{i+1} and then instantaneously increases to value V_{i+1} (C_{i+1}). We can then replace the integral $\int_0^t e^{d_L \tau} V(\tau) C(\tau) d\tau$ with the sum $1/d_L \sum_{i=1}^{n} V_i C_i (e^{d_L t_{i+1}} - e^{d_L t_i})$. For the transplant patients [**?**], assume that values below the detection limit of ultrasensitive assays at a single time point are equal to 0.5 copies per ml (the minimum detectable value), while second and subsequent undetectable values are assumed to be zero. Values below the standard clinical assay were taken to be equal to 5 copies per ml. Hence for Patient A, the sequences are $\{V_i\}$ = { 0.5, 104, 0.5, 65, 0.5, 0, 0, 0}, $\{C_i\}$ = { 800, 800, 600, 600, 270, 270, 900, 1050}, and t_i = {0, 29, 64, 143, 216, 587, 1075, 1266}. For Patient B, the sequences are $\{V_i\}$ = { 0.5, 0, 0, 5, 0.5, 0.5, 0, 0, 0}, $\{C_i\}$ = { 1050, 1050, 800, 600, 600, 400, 400, 500, 870} and t_i is {0, 59, 92, 150, 281, 410, 519, 652, 949}. The resulting estimates for reservoir size are 11 [1.1, 110] $\times 10^{-3}$ IUPM for Patient A and 57 [5.7, 570] $\times 10^{-4}$ IUPM for Patient B.

Method 2:

Longitudinal studies tracking the movement of particular unique viral genetic sequences between plasma virus and resting memory $CD4^+$ T cells have directly estimated the rate of entry into the LR during cART [**?**]. By scaling this entry rate by the patient's viral loads and CD4 count (reported in [**?**]), we can calculate the entry rate per plasma virus and per CD4 cell (E). We can then use this entry rate, along with the observed plasma virus levels and CD4 counts in the Boston patients, as

another method to estimate the amount of new latently infected donor cells that could have been created during transplant. Similar to S2 Text Eq. 2, with this empirically observed entry rate we get a predicted LR size after time t of

$$L_{pred}(t) = Ee^{-d_L t}\int_0^t e^{d_L \tau} V(\tau)C(\tau)d\tau, \tag{3}$$

where again we can replace the integral $\int_0^t e^{d_L\tau}V(\tau)C(\tau)d\tau$ with the sum $1/d_L \sum_{i=1}^{n} V_i C_i(e^{d_L t_{i+1}} - e^{d_L t_i})$ to account for discrete sampling times.

We summarize here how E is estimated. Sedaghat et al [?] tracked specific viral genetic sequences (termed "predominant plasma clones", or PPCs) in the plasma and in resting CD4$^+$ T cells in order to calculate the entry rate into the reservoir during cART. The maximum likelihood estimate for the reservoir entry rate (cells per day), are reported for their three patients in Table **??**. We augment this analysis by translating these rates into levels per plasma viral load level and CD4 level, so that we can extract the parameter E and apply this to other patients who have different potential for ongoing replication and entry (Table **??**). These values are reported in another study using the same patient data[?]. We calculated the average viral load for each patient over the course of monitoring. Nettles et al [?] found viral loads less than the assay detection limit of 50 copies per ml on $> 90\%$ of tri-weekly measurements for Patients 135 and 148 and $\approx$60% in Patient 154. Other work using ultra-sensitive viral single-copy RNA assays on patients consistently suppressed below 50 copies per ml on cART found that the average residual viral load was 5 copies per ml [?], and so we used this value for all points in this range when calculating the average. The only reported CD4 count was at the beginning of the study (at which point patients had already been suppressed on cART for at least 6 months), and we assumed this level was constant throughout the period of monitoring.

Table S3.1. Data used to calculate the entry rate of cells into the latent reservoir during cART. Sedaghat et al [?] tracked particular viral genetic sequences in the plasma and in latently infected cells to determine the rate at which new cells entered the reservoir. Their results for three patients are shown (MLE: Maximum likelihood estimate). The average viral load during the study period, and the CD4 count at the beginning of the study, were derived from Nettles et al. [?] as described in the text.

Patient	Entry rate, MLE (cells d^{-1})	Avg. viral load (RNA copies/ml)	CD4 count (cells/ul)	Scaled entry rate (E) (cells d^{-1} copies^{-1} ml cells$^-$1 ul)
135	0	7	445	0
148	0	5	670	0
154	44	30	167	0.0088
Average				0.003

There are many limitations to this approach. The limited number of sequences sampled means

that this method could only detect a change in reservoir composition of around 1%. Consequently, they cannot detect entry rates less than around 30 cells per day. Even this level would be quite high in treated patients, given that in untreated patients, in whom viral loads (and presumably by extension reservoir entry rates) are about 10^4 times as high, the reservoir size does not change dramatically beyond acute infection. This detection limit is reflected in the fact that for two patients they were unable to observe any entry into the reservoir, leading to a maximum likelihood entry rate of 0 cells per day (although the confidence intervals they report are quite large). The assumption behind the sequence tracking method is that the PPC sequence is initially absent from the reservoir, though it could have been present but not sampled. This would lead to inflation of the entry rate estimates, which could have occurred in patient 154.

Using the average entry rate (E) over the three patients for a central estimate, and the minimum and maximum values for ranges, we estimate the number of newly infected donor cells in Patient A to be 8000 [0, 24 000] , and in Patient B to be 970 [0, 2900].

We can compare the the results from both methods by using a conversion between IUPM (which Method 1 returns) and number of infected cells (which Method 2 returns). We do this by assuming a total of 10^{12} resting CD4$^+$ T cells (the denominator for IUPM). The range suggested by Method 2 is completely contained within the range from Method 1, so we report the latter.
